# Editorial: Design, Synthesis, and Application of Novel π-Conjugated Materials

**DOI:** 10.3389/fchem.2020.634698

**Published:** 2021-01-11

**Authors:** Zhicheng Dai, Taotao Ai, Qixin Zhou, Haichang Zhang

**Affiliations:** ^1^Key Laboratory of Rubber-Plastics of Ministry of Education/Shandong Province (QUST), School of Polymer Science & Engineering, Qingdao University of Science & Technology, Qingdao, China; ^2^National and Local Joint Engineering Laboratory for Slag Comprehensive Utilization and Environmental Technology, School of Material Science and Engineering, Shaanxi University of Technology (SNUT), Hanzhong, China; ^3^National Center for Education and Research on Corrosion and Materials Performance, Department of Chemical and Biomolecular Engineering, The University of Akron, Akron, OH, United States

**Keywords:** π-conjugated materials, electronics, OPV, OLED, sensor

In recent years, extensive research efforts have been dedicated to the development of novel π-conjugated materials, including small molecules, oligomers, and polymers. These materials have been adapted to various applications, such as in organic-field effect transistors (OFET), organic photovoltaic (OPV) sensors, and organic light emitting diodes (OLED), etc. Among these, π-Conjugated materials with various applications have been designed with specific molecular concepts in mind. This Research Topic includes 12 articles outlining original research and reviewing and describing a series of novel π-conjugated materials with various applications. The articles provide an overview of different types of π-conjugated materials and how they are designed and characterized, providing an overview of progress and development in this field.

Among the articles selected for this Research Topic, seven are original research articles. The development of high-sensitivity naked-eye detection sensors for fluoride anions is crucial and challenging because fluoride anions play a key role in human health and chemical engineering. Yuan et al. reported a new type of colorimetric chemosensor dye based on indaceno(1,2-b:5,6-b′)dithiophene]-2,7-diylbis(methanylylidene) bis(indolin-2-one) (IDTI). IDTI can interact with fluoride anions to exhibit color changes that are visible to the naked eye, with changes occurring from red to orange or yellow and from purple to green or blue under ambient light and 365-nm UV light, respectively. The spectroscopic studies indicated that IDTI could be used to quantitatively analyze the fluoride concentration with a detection limit as low as 1 ×10^−7^ M, which is one of the highest sensitivity fluoride sensors reported (Yuan et al.). Another research paper describes how Deng's group developed a simple compound based on aminobenzodifuranone (ABDF) to detect fluoride anions. ABDF responds to only the fluoride anion among various anions and exhibits a color change from dark blue to various colors (colorless, yellow, orange, and red) in different common organic solvents. This work provides new insights into the development of highly sensitive and selective fluoride chemosensors, and the functionality required to detect the polarity of the solvents (Deng et al.).

The structure-performance relationship of conjugated polymers has drawn significant attention. Frizon et al. prepared and characterized a series of novel π-conjugated fluorescent materials based on derivatives of 2,1,3-benzoxadiazole (BTD). The photophysical behavior of the compounds was investigated using spectroscopic techniques (Frizon et al.). In another study included in this Research Topic, Lin et al. synthesized six conjugated polymers using a similar polymer backbone with different numbers of thiophene units, using electrochemical polymerization, showing that increasing the π-conjugated length could significantly improve the electrochemical stability of polymers.

Thermally activated delayed fluorescent (TADF) materials have been considered as one of the most promising candidates in OLED because of their capability in harvesting all the triplet excitons. However, designing high performance TADF materials is challenging. Jin and Xin designed and investigated a series of π-conjugated materials with a donor-acceptor system. The authors discovered that efficient separation could be observed between donor and acceptor fragments: a rational design for efficient TADF materials and charge transport materials in OLED applications (Jin and Xin).

Synthesized polypeptide is a chemical component and secondary structure to a natural protein that plays a key role in bio-related fields. However, polypeptide exhibits no switching properties and shows no benefits as a potential control component. Hu's group synthesized and characterized light-responsive poly(γ-benzyl-L-glutamate)s using a simple one-step NCA method, which could facilitate the trans → cis transition and shorten the recovery time of the cis → trans process under different conditions (Chen et al.).

Tautomerism has been an active area of research over the past few decades. Upon investigation of a series of G·C nucleobase pairs for the first time, Brovarets's group established that the G·C base pair is incorporated into the DNA/RNA double helix with parallel strands in the form of the G^*^·C*_O2_${\text{C}}_{\text{O}2}^{*}$(rWC), G·C^*^(rw_WC_), and G^*^·C(rw_WC_) tautomers, which are in rapid tautomeric equilibrium with each other (Brovarets' et al.).

This Research Topic also includes five review articles. Diketopyrrolopyrrole (DPP) and its derivatives as an electron-deficient unit have been developed in various applications and attracted significant attention in recent years due to its distinctive properties. The Research Topic also includes a study by Deng et al. on the use of DPP-based materials in OFETs, OPVs, sensors, two-photon absorption, and coatings, etc. In addition, a prospective study provides insights into ways of further functionalizing DPP and its derivatives, such as enlargement of π-conjugation, modification of alkyl chain, design of high-performance polymers for the promotion in π-conjugated materials applications (Bao et al.).

Supramolecular polymeric materials are widely used in many fields and have been rapidly progressing in the past decade. From the perspective of the terpyridine-based polymers, Liu et al.not only summarized the development of some novel molecules in the field of energy conversion but also highlighted the Zn(II)- and Ru(II)-containing supramolecular polymers in light-emitting materials and photovoltaic materials. This review also showed that terpyridine-based materials with easy modification, organic/inorganic hybrid, definite structure, etc., open a new pathway to construct novel structures with interesting properties in photoelectric materials (Liu et al.).

Ionochromism is realized through the coordination between metal and ligand, resulting in sensitivity to metal ions, which could be used as metal sensors. Liu et al. reviewed not only an overview of the developments of ionochromic effect according to the type of ligands, but also highlighted the mechanism of ionochromism, metal recognition sensitivity, and their application in sensors. Additionally, the authors pointed out that the preparation of chemical sensors with high sensitivity, long-term stability, and selectivity is still a critical challenge. This review provides a rational structure design and evaluation of chemosensors. Apart from that, π-conjugated materials with semiconductor behavior are regarded as promising spin-transport materials due to the weak spin-orbit coupling interaction and hence long spin relaxation time (Liu et al.). Zhang et al. reviewed organic semiconductors from the perspectives of spin transport, spin functional devices, all-organic spin devices, spin manipulation, and then summarized their challenges. Furthermore, the authors observe that various organic semiconductors need to be tested and that the strategic step design of semiconductors should be explored to overcome the present challenges (Zhang et al.).

OPV is one of the most promising energy generation technologies. To build high performance OPV, the crystalline control of the materials is crucial. However, this topic has yet to attract enough attention in the scientific and industrial community. In their contribution, Qiu et al. review the structure and crystallinity of the representative polymer donor materials and corresponding device properties. Several typical methods for controlling the crystallinity of materials were also addressed. The author claimed that only the effort of all the scientists in terms of materials design, theoretical research, device optimization, and OSCs could then be widely used in everyday life, with exceptional market prospects in the next few years (Qiu et al.).

The 12 research articles included here indicate that the optical and electrochemical properties, solubility, charge transferability, and other intrinsic physical and chemical properties of materials could be easily controlled by adjusting molecular structures. Thus, the molecular design concept plays a critical role in high performance π-conjugated materials and their potential applications.

## Author Contributions

ZD prepared the manuscript. TA, QZ, and HZ revised the manuscript and are topic editors of this Research Topic. All authors contributed to the article and approved the submitted version.

## Conflict of Interest

The authors declare that the research was conducted in the absence of any commercial or financial relationships that could be construed as a potential conflict of interest.

